# Rice-Based Diet and Cardiovascular Disease Mortality in Japan: From the Takayama Study

**DOI:** 10.3390/nu14112291

**Published:** 2022-05-30

**Authors:** Keiko Wada, Shino Oba, Chisato Nagata

**Affiliations:** 1 Department of Epidemiology and Preventive Medicine, Graduate School of Medicine, Gifu University, Gifu 501-1193, Gifu, Japan; oba@gunma-u.ac.jp (S.O.); chisato@gifu-u.ac.jp (C.N.); 2 Graduate School of Health Sciences, Gunma University, Maebashi 371-8514, Gunma, Japan

**Keywords:** grain food, rice, Japanese diet, cardiovascular disease, mortality, cohort studies, epidemiology

## Abstract

Rice is the staple food in Japan and many other Asian countries, but research on rice-based diets and cardiovascular disease is limited. We aimed to evaluate the association between rice consumption as grain dishes and cardiovascular disease mortality in comparison with bread and noodle consumption. The subjects were 13,355 men and 15,724 women aged ≥35 years who enrolled in the Takayama Study. Diet intake was assessed using a validated food-frequency questionnaire. Causes of death were identified from death certificates. Cardiovascular disease was defined according to the International Classification of Diseases and Health Related Problems, 10th Revision (code I00–I99). Hazard ratios in the second, third, and highest quartiles versus the lowest quartile of rice intake for cardiovascular disease mortality were 0.98, 0.80, and 0.78 for men, respectively (trend *p* = 0.013), but no significant association was observed among women. Rice intake was positively correlated with the intake of soy products and seaweed, and negatively correlated with the intake of meat and eggs. Neither bread nor noodles were associated with cardiovascular disease mortality. In Japan, choosing rice as a grain dish is likely to be accompanied by healthier foods as side dishes, which may have a potential role in the prevention of cardiovascular disease.

## 1. Introduction

The Japanese diet is believed to contribute to longer life expectancy and lower morbidity from cardiovascular disease, particularly coronary heart disease, in Japanese people compared with people in Western countries [1]. Rice is the staple food of the Japanese diet, and a typical Japanese meal consists of rice as a grain dish along with soup and several other dishes. In recent years, with the Westernization of the Japanese diet, rice consumption has been decreasing while bread consumption has been increasing [2,3].

The intake of whole grains is associated with reduced incidence and risk of mortality from cardiovascular disease, whereas no significant associations have been found between intake of refined grains and cardiovascular disease [4,5]. In Japan, the most commonly consumed rice is refined white rice, not brown rice. Rice consumption is much higher in Asia than in Western countries [6], and a positive association between rice consumption and risk of diabetes has been found primarily in the Asian population [6,7,8]. A pooled analysis of three US cohorts showed that consumption of white or brown rice was not associated with cardiovascular disease risk [9]. In Asian cohorts (Chinese and Japanese), three studies observed null associations between rice consumption and ischemic heart disease mortality [10], cardiovascular disease incidence and mortality [11], and stroke mortality [12], while two studies observed an inverse association with cardiovascular disease mortality [13,14]. In a cohort study conducted in 21 countries in different regions of the world, no significant associations were observed between rice intake and cardiovascular disease incidence and mortality [15]. Most studies to date have been concerned with the health risks associated with rice intake itself and have attempted to demonstrate an association between rice intake and cardiovascular disease independent of the intake of other foods, for example, by adjusting for intakes of fruit and vegetables [10,11,13,14,15], dairy products [13,15], eggs [10], fish [10,11,13], legumes (including soybeans) [10,11,13], and meat [10,11,13,14,15]. In Western countries, refined grains are a large part of an unhealthy dietary pattern that also includes red and processed meats, sugar-sweetened foods and beverages, French fries, and high-fat dairy products, and the health risks associated with refined grain intake are thought to be largely due to the association with these other foods rather than the refined grains themselves [5].

The Japanese Food Guide Spinning Top, which is widely used in dietary health guidance, illustrates the balance and quantity of food in the daily Japanese diet [16]. This food guide is based on the intake of grain dishes, vegetable dishes, fish and meat dishes, milk, and fruits, as well as energy from snacks and alcoholic beverages. Grain dishes are at the top part of the spinning top, which means they are the most essential component of the diet for supplying energy. Grain dishes include the rice, bread, and noodle dishes that are commonly consumed in Japan. When Japanese people choose a grain dish for a meal, they are likely to choose side dishes that match well with it. In other words, the choice of grain dishes may determine what other foods are eaten together; for example, in the traditional Japanese diet, steamed white rice is often eaten together with miso soup, which is made from soybean paste. Thus, it may be important to investigate the role that rice intake plays in cardiovascular disease as well as associated food intake patterns.

In this population-based prospective study in Japan, we aimed to evaluate the association between the consumption of rice as a grain dish and cardiovascular disease mortality in comparison with consumption of bread and noodles as grain dishes, and to describe the food intake patterns associated with these three common grain dishes in Japan.

## 2. Materials and Methods

### 2.1. Participants and Design

The subjects were participants in a cohort study in Japan, the Takayama Study. This cohort has been followed in order to study the associations of lifestyle factors with the risk of mortality from cancer and other diseases. The details of this study have been described elsewhere [17]. Briefly, the target population was 36,990 non-hospitalized residents who were 35 years of age or older in September 1992; 31,552 participated in the study and completed a self-administered questionnaire that included a food frequency questionnaire (FFQ) (response rate, 85.3%). Questions included demographic characteristics, medical history, physical activity, smoking status, and reproductive factors.

### 2.2. Food Consumption and Other Exposure Variables

The FFQ consisted of questions regarding the consumption frequency of various foods and dishes (169 items) and the usual portion size of meals during the past year [18]. Based on this information, the intake of each nutrient and food group was estimated using the Japanese Standard Table of Food Composition, 5th revised and enlarged edition [19]. The Spearman correlation coefficients between the FFQ and 12 one-day diet records kept over a 1-year period for total energy, carbohydrate, protein, and fat intake were, respectively, 0.44, 0.34, 0.38, and 0.24 for men and 0.53, 0.45, 0.63, and 0.52 for women. The coefficients for food groups ranged from 0.18 (meat) to 0.90 (milk and dairy products) in men, and from 0.33 (seafood) and 0.77 (milk and dairy products) in women. In this study, we focused on three grain dishes common in Japan: rice, bread, and noodles. Rice consumed as a grain dish included cooked white rice, half-glutinous rice, brown rice, rice balls, sushi (nigiri sushi, chirashi sushi, inari sushi, and sushi rolls), seasoned rice, rapeseed rice, fried rice, red rice (steamed rice with red beans), and rice porridge. Bread consumed as a grain dish included sliced bread, rolls, buns, French bread, whole grain bread, rye bread, sandwiches, and pizza. We excluded sweet breads (bread with sweetened red bean paste, cream, or jam), Danish pastries, pancakes, and doughnuts, which are commonly consumed as snacks in Japan. Noodles consumed as a grain dish *included udon, soba, ramen*, spaghetti, and gratin. Rice, wheat, bread, and noodles included as ingredients in fish, meat, and vegetable dishes as well as in confectionaries were excluded from the calculation of grain dish intakes.

Smokers were defined as persons who had smoked a total of 20 or more packs of cigarettes during their lifetime. Those with a history of smoking were also asked about the number of years they had smoked. Physical activity was assessed by asking the participants how much time, on average, they had spent engaged in physical activity of various intensities during the previous year. The time spent at each intensity level of activity (hours/week) was multiplied by the corresponding energy expenditure expressed as a metabolic equivalent (MET), and the total score of the product was taken as the physical activity score (MET·h/week). The details of the physical activity assessment, including its validity, have been reported elsewhere [20].

### 2.3. Cardiovascular Disease Mortality and Follow-Up

After excluding 1072 men and 1401 women who reported in the baseline questionnaire that they had a history of cancer or cardiovascular disease (coronary heart disease and stroke), 13,355 men and 15,724 women were included in the analysis. Information on deaths and emigrations from baseline to 1 October 2008 was obtained from residential or family registers. The causes of death, identified from death certificates provided by the Legal Affairs Bureau, were coded according to the International Classification of Diseases and Health Related Problems, 10th Revision. Cardiovascular disease was defined as code I00–I99. Follow-up was conducted until the date of death, date of moving out of the study area, or 1 October 2008, whichever came first. During the average follow-up period of 14.1 years, 1769 persons (6.1%) moved out of the study area. For the 229 (0.8%) whose move-out date was unknown, the date of last residence in the study area was assigned as the censoring date.

### 2.4. Statistical Analysis

The intakes of grains and other foods were adjusted for energy intake by using the residual method proposed by Willett [21]. The study participants were classified into quartile groups (Q1, Q2, Q3, or Q4) according to their energy-adjusted intake of rice, bread, and noodles as grain dishes. Participant characteristics were calculated as the mean (standard deviation) or number (percentage) of each category according to the quartile groups of grain-dish rice intake by sex.

To compare food intake patterns related to each of the three grain intakes, Spearman’s correlation coefficients were calculated, controlling for age and total grain intake, between intakes of rice, bread, or noodles and intakes of other foods, including potatoes, meat, seafood, soy products, vegetables, seaweed, fruits, fungi, eggs, milk and dairy products, and confectionaries.

A Cox proportional hazards model was used to estimate the hazard ratios and 95% confidence intervals (CIs) of cardiovascular disease mortality for the quartile groups of intakes of rice, bread, and noodles. The reference group was defined as the lowest quartile of each grain food intake. Potential cardiovascular risk factors identified by a literature review were adjusted as the following confounders: age (years, continuous), marital status (yes, no), years of education (≤8, 9–11, 12–14, ≥15 years), body mass index (quartiles), smoking status (never, former, current smoker who had smoked for ≤30 years, current smoker who had smoked for ≥31 years), physical activity score (MET·h/week, continuous), history of diabetes and hypertension (yes, no), alcohol consumption (quartiles), coffee consumption (none, <1 cup/day, ≥1 cup/day), and salt intake (quartiles). For women, menopausal status (premenopausal, postmenopausal) was also adjusted. Because our interest was mainly in grain dishes and accompanying foods, we initially avoided adjusting for the consumption of other nutrients or foods except for alcohol, coffee, and salt. Dummy variables were created for missing data in categorical covariates. Tests for linear trends were performed using the median values of grain intakes for each category.

A sensitivity analysis was performed excluding deaths during the first 2 years of follow-up, considering the potential influence of physical condition on diet at baseline. All analyses were performed using SAS version 9.4 (SAS Institute, Cary, NC, USA). *p*-values were calculated by two-tailed tests; *p*-values less than 0.05 were considered statistically significant for all analyses.

## 3. Results

During the study period, 778 men and 907 women died of cardiovascular disease. The characteristics of the participants are shown according to the quartile groups of energy-adjusted rice intake in Table 1. Men with lower rice intake were more likely to have a history of diabetes or hypertension, be less physically active, and have higher alcohol consumption and higher intake of fiber and salt. Women in the lowest quartile of rice intake had higher levels of education, higher alcohol and coffee consumption, and higher intake of fiber and salt compared with women in the higher quartiles.

For both men and women, rice intake was positively correlated with intake of soy products and seaweed, and was more negatively correlated with the intake of meat and eggs than the others (Table 2). The intake of bread was more positively correlated with the intake of fruit and dairy products and negatively correlated with the intake of soy products. The intake of noodles was more positively correlated with the intake of potatoes, meat, seafood, and eggs. In addition, the Spearman correlation coefficients after adjustments for age were −0.21 between rice and bread, −0.25 between rice and noodles, and −0.02 between bread and noodles in men, and −0.26 between rice and bread, −0.18 between rice and noodles, and −0.03 between bread and noodles in women.

Compared with men in the lowest quartile of rice intake, men in the second, third, and highest quartiles had decreased risks of cardiovascular disease mortality with a significant linear trend (hazard ratios, 0.98, 0.80, and 0.78, respectively; trend *p* = 0.013), whereas no significant association was observed among women (Table 3 and Table 4). The intakes of bread and noodles were not associated with cardiovascular disease mortality in either men or women.

We also analyzed the association between rice intake and mortality from coronary heart disease and stroke in men (Table 5). Higher rice consumption was associated with lower mortality risk for both coronary heart disease (trend *p* = 0.046) and stroke (trend *p* = 0.049).

To further evaluate the association between rice intake and cardiovascular disease independent of the intake of soy products, seaweed, meat, and eggs, we additionally adjusted for these food intakes (quartiles). The results were largely unchanged, with hazard ratios of 0.99 (95% CI: 0.82–1.19), 0.81 (95% CI: 0.64–1.02), and 0.78 (95% CI: 0.62–1.00) for male cardiovascular disease mortality in the second, third, and highest quartiles, respectively, versus the lowest quartile of rice intake (trend *p* = 0.018). However, the risk reductions for coronary heart disease and stroke with rice intake were somewhat weaker; the hazard ratios for the second, third, and highest quartiles versus the lowest quartile of rice intake were, respectively, 1.00 (95% CI: 0.66–1.53), 0.68 (95% CI: 0.40–1.15), and 0.68 (95% CI: 0.39–1.17) for coronary heart disease (trend *p* = 0.080) and 0.84 (95% CI: 0.63–1.12), 0.79 (95% CI: 0.55–1.13), and 0.76 (95% CI: 0.52–1.10) for stroke (trend *p* = 0.13).

In addition, when we additionally adjusted for the score of adherence to the Japanese Food Guide Spinning Top [16], the association between rice intake and cardiovascular disease was not substantially changed; the hazard ratios for male cardiovascular disease mortality were, respectively, 0.98 (95% CI: 0.81–1.18), 0.79 (95% CI: 0.63–1.00), and 0.77 (95% CI: 0.61–0.98) for the second, third, and highest quartiles versus the lowest quartile of rice intake (trend *p* = 0.012). When we adjusted for hypertension medication use instead of history of hypertension, the corresponding hazard ratios for male cardiovascular disease mortality were, respectively, 1.00 (95% CI: 0.83–1.21), 0.81 (95% CI: 0.64–1.03), and 0.79 (95% CI: 0.62–1.01) (trend *p* = 0.022).

When we re-analyzed the data after excluding deaths during the first 2 years of follow-up (250 men and 203 women), the hazard ratio of male cardiovascular disease mortality for the highest quartile versus the lowest quartile of rice intake was 0.77 (95% CI: 0.60–0.98) (trend *p* = 0.012).

## 4. Discussion

In this prospective study conducted in Japan, we found that higher consumption of rice as grain dishes was significantly associated with a decreased risk of cardiovascular disease mortality in men. Neither bread nor noodle intake as grain dishes was associated with cardiovascular disease mortality.

Compared with bread and noodles, rice consumed as grain dishes was associated with higher intakes of soy products and seaweed and lower intakes of meat and eggs. Because an additional adjustment for these food intakes weakened its association with coronary heart disease and stroke, the reduced risk of mortality in these two diseases related to rice intake may be due in part to the association with these food intakes. The intake of soy products and seaweed is associated with decreased risk of cardiovascular disease and related risk factors, especially in Asian countries [22,23,24,25]. In contrast, the consumption of red and processed meat is associated with increased risk of cardiovascular disease [26,27], although there is no conclusive evidence for the role of egg intake in cardiovascular disease risk [28]. In addition, the significant association between greater rice intake and decreased cardiovascular disease mortality remained after making additional adjustments for the intake of these foods, suggesting that rice itself might be protective against cardiovascular disease. Rice has a high starch content and a high intake of starch is associated with lower cardiovascular disease mortality in men in this cohort [29]. Rice also contains some fiber and vitamins. Dietary fiber and vitamin B6 have been reported to have negative associations with coronary heart disease and heart failure in the Japanese population [30,31,32].

In contrast, no significant association between rice consumption and cardiovascular disease mortality was observed in women. The reasons are unclear, but confectionary intake was negatively associated with rice intake for men and positively associated with rice intake for women, which is different compared to most foods whose intake patterns were associated with rice intake similarly in both men and women. In addition, the positive association between rice consumption and diabetes was greater in women than in men [7,33]. A sex difference in lipoprotein metabolism in response to dietary intake has been suggested, and a greater increase in triglycerides and a lesser decrease in low-density lipoprotein (LDL) cholesterol have been observed in women compared with men consuming a high-carbohydrate diet [34]. Diabetes and dyslipidemia are known risk factors for cardiovascular diseases. Lastly, we observed an increased risk of cardiovascular mortality related to a high glycemic index, which is primarily due to rice consumption, in women but not men [35].

The role of bread and noodle intakes on cardiovascular disease in the Asian diet has not been thoroughly investigated. A bread-and-dairy dietary pattern is associated with higher LDL and total cholesterol [36], higher fasting glucose [37], and lower C-reactive protein levels [38]. Noodle intake or a noodle dietary pattern was associated with higher high-density lipoprotein and total cholesterol [36], higher hemoglobin A1c [39,40], and higher phosphorus levels [41]. Although principal component analysis has been used to derive various dietary patterns in Japan, some patterns varied considerably in terms of contents across studies and their reproducibility was low; one reproducible dietary pattern was characterized by a higher intake of mushrooms, seaweed, vegetables, potatoes, fruits, pulses, and pickles [42]. Dietary patterns not derived by principal component analysis, including the grain dishes investigated in the present study, would also have an impact on cardiovascular health. Further research may be warranted to investigate the role of dietary patterns in a country-specific hypothesis-derived approach as well as a data-derived approach.

The merits of our study include its prospective design, good participation rate, long follow-up period, and consideration of several confounding factors.

In this study, as in most epidemiological studies, a questionnaire was used to collect participants’ information. The FFQ was validated for various types of foods listed, but it was designed to measure an individual’s relative intake of foods and nutrients rather than the absolute values; accordingly, the estimates of rice intake derived from the FFQ were higher than the estimates based on 12-day dietary records. However, measurement errors by such misclassifications are likely to be independent of death. The composition of some foods or dishes in our FFQ, for example, rice with nori (brown seaweed) for rice balls, and noodles with pork for chashu (roasted pork fillet) on ramen noodles, and so on, might have influenced the observed correlations between intakes of grain dishes and other foods. However, all meals in our FFQ were based on the typical diet for Japanese people.

Several other limitations should be mentioned. Although we considered history of hypertension and diabetes in the analysis, we could not exclude the possibility of residual confounders by other disease such as dyslipidemia. We also did not obtain the information on medication use for diabetes or other chronic diseases. The information on exposure, including the FFQ, was collected only at baseline, and thus changes in lifestyle habits were not evaluated during the follow-up period. Dietary patterns and lifestyle habits might have changed due to preclinical signs or underlying disease. However, the exclusion of deaths during the first 2 years of follow-up did not change the results significantly. Lastly, the findings regarding diet related to rice may be generalizable to a limited population with similar dietary habits and culture. Because the Japanese diet has become increasingly Westernized [3], caution should be exercised when interpreting the results. However, because grain dishes are easily understood in the context of dietary advice in Japan, our findings may be of interest to health providers.

## 5. Conclusions

This prospective study observed an association between higher consumption of rice as grain dishes and decreased cardiovascular disease mortality in men, which may be largely attributable to the intake of rice itself as well as in part to the accompanying foods. In contrast to the Western diet, in which the intake of refined grains can lead to unhealthy eating patterns, choosing rice as a grain dish in Japan is likely to be accompanied by consumption of healthier foods as side dishes, which may contribute to the prevention of cardiovascular disease.

## Figures and Tables

**Table 1:** Characteristics of study participants at baseline according to the quartiles (Q1–Q4) of intake of rice as a grain dish.

	Rice Intake									
	Men					Women				
	Q1	Q2	Q3	Q4	*p*	Q1	Q2	Q3	Q4	*p*
Number of participants	3339	3339	3339	3338		3931	3931	3931	3931	
Age (years)	54.3 (12.5)	55.9 (13.2)	51.7 (10.7)	54.1 (11.6)	<0.01	53.3 (11.9)	54.7 (13.0)	58.7 (14.2)	53.9 (12.2)	<0.01
Body mass index (kg/m^2^)	22.5 (2.8)	22.4 (3.0)	22.6 (2.6)	22.4 (2.7)	<0.01	22.0 (2.7)	22.0 (2.9)	21.9 (3.1)	22.0 (2.9)	<0.01
History of diabetes (yes, %)	7.9%	6.8%	4.9%	4.3%	<0.01	2.9%	2.9%	3.3%	1.6%	<0.01
History of hypertension (yes, %)	20.7%	19.7%	18.4%	16.9%	<0.01	15.7%	18.1%	20.0%	15.6%	<0.01
Menopausal status (pre)	-	-	-	-		45.2%	43.0%	32.4%	43.9%	<0.01
Marital status (yes, %)	91.3%	90.5%	93.4%	90.5%	<0.01	76.4%	77.3%	68.0%	79.1%	<0.01
Education years (%)					<0.01					<0.01
≤8 years	22.8%	28.9%	20.6%	27.7%		17.8%	25.7%	37.3%	25.9%	
9–11 years	22.1%	23.3%	26.4%	28.2%		40.1%	38.3%	36.5%	43.3%	
12–14 years	26.9%	24.4%	26.0%	22.7%		35.0%	31.4%	22.9%	27.2%	
≥15 years	15.8%	23.7%	12.6%	7.6%		7.1%	4.7%	3.3%	3.6%	
Physical activity score(METs·h/week)	24.8 (38.4)	26.6 (40.0)	30.1 (42.3)	29.8 (44.5)	<0.01	19.9 (28.9)	20.1 (29.7)	17.2 (28.4)	20.2 (30.6)	<0.01
Smoking status (%)					<0.01					<0.01
never	24.7%	27.2%	23.3%	24.8%		79.6%	83.3%	84.4%	82.8%	
former	26.4%	26.1%	23.6%	23.9%		4.9%	4.5%	4.4%	3.8%	
current (for <30 years)	24.4%	22.6%	28.4%	24.7%		14.0%	10.7%	8.9%	11.3%	
current (for ≥30 years)	24.3%	25.1%	23.7%	27.0%		1.6%	1.5%	2.3%	2.1%	
Alcohol consumption (g/day)	54.5 (46.9)	42.5 (44.9)	43.2 (35.4)	27.7 (32.4)	<0.01	12.2 (23.2)	6.7 (13.1)	5.6 (14.4)	6.4 (13.6)	<0.01
Total energy intake (kcal/day)	2668 (934)	2557 (1157)	2775 (463)	2486 (768)	<0.01	2488 (822)	1859 (431)	1849 (984)	2334 (561)	<0.01
Carbohydrate intake (g/day)	328 (118)	345 (149)	397 (66)	388 (103)	<0.01	334 (114)	266 (61)	278 (137)	374 (77)	<0.01
Fiber intake (g/day)	18.4 (10.3)	16.7 (10.0)	16.0 (6.4)	13.6 (6.9)	<0.01	21.8 (10.8)	15.2 (5.6)	14.3 (9.5)	15.4 (6.6)	<0.01
Salt intake (g/day)	16.4 (6.9)	14.6 (7.4)	14.1 (4.4)	11.6 (5.4)	<0.01	16.9 (6.5)	12.1 (3.7)	11.2 (6.6)	12.1 (4.6)	<0.01
Coffee consumption					<0.01					<0.01
none	18.6%	23.5%	16.1%	21.1%		18.0%	23.3%	36.9%	27.4%	
<1 cup /day	36.1%	37.5%	38.9%	42.7%		36.4%	40.7%	37.3%	42.2%	
≥1 cup /day	45.3%	39.0%	45.0%	36.2%		45.6%	36.0%	25.8%	30.5%	
Mean (standard deviation) or percentage										

**Table 2:** Spearman correlation coefficients between intakes of rice, breads, and noodles as grain dishes with intakes of other foods.

	Rice		Bread		Noodles	
	ρ ^a^	*p*	ρ ^a^	*p*	ρ ^a^	*p*
**Men**						
Potatoes	−0.090	<0.001	0.115	<0.001	0.127	<0.001
Meat	−0.137	<0.001	0.101	<0.001	0.223	<0.001
Seafood	−0.050	<0.001	−0.056	<0.001	0.159	<0.001
Soy products	0.071	<0.001	−0.101	<0.001	−0.014	0.096
Vegetables	−0.067	<0.001	0.077	<0.001	0.053	<0.001
Seaweed	0.048	<0.001	−0.027	<0.001	−0.005	0.54
Fruits	−0.073	<0.001	0.236	<0.001	0.019	0.032
Fungi	−0.025	0.003	0.071	<0.001	0.080	<0.001
Eggs	−0.116	<0.001	0.052	<0.001	0.105	<0.001
Milk, dairy products	−0.110	<0.001	0.269	<0.001	−0.025	0.003
Confectionaries	−0.015	0.080	0.147	<0.001	0.070	<0.001
**Women**						
Potatoes	−0.038	<0.001	0.028	<0.001	0.132	<0.001
Meat	−0.105	<0.001	0.027	<0.001	0.250	<0.001
Seafood	−0.038	<0.001	−0.053	<0.001	0.160	<0.001
Soy products	0.070	<0.001	−0.122	<0.001	0.009	0.24
Vegetables	−0.057	<0.001	0.008	0.29	0.062	<0.001
Seaweed	0.035	<0.001	−0.043	<0.001	0.006	0.42
Fruits	−0.047	<0.001	0.128	<0.001	0.026	0.001
Fungi	−0.014	0.085	0.027	<0.001	0.086	<0.001
Eggs	−0.102	<0.001	0.035	<0.001	0.112	<0.001
Milk, dairy products	−0.050	<0.001	0.159	<0.001	−0.087	<0.001
Confectionaries	0.021	0.008	0.084	<0.001	0.105	<0.001

Food intakes were adjusted for energy intake by Willet method. ^a^ Correlation coefficient ρ partially controlling for age and total grain intake.

**Table 3:** Hazard ratios of cardiovascular disease mortality among Japanese men according to the quartiles (Q1–Q4) of intakes of rice, breads, and noodles as grain dishes.

	Median Intake (g/day)	No. of Subjects	Person-Years	No. of Deaths	Age-Adjusted		Multivariate-Adjusted ^a^	
					HR	95% CI	HR	95% CI
Rice								
Q1	153.4	3339	44,442	224	1.00	ref	1.00	ref
Q2	210.5	3339	44,108	257	1.03	(0.86–1.23)	0.98	(0.81–1.18)
Q3	286.1	3339	47,832	127	0.77	(0.62–0.96)	0.80	(0.63–1.01)
Q4	321.5	3338	47,056	171	0.81	(0.66–0.99)	0.78	(0.62–0.99)
trend *P*						0.004		0.013
Bread								
Q1	2.9	3339	47,223	175	1.00	ref	1.00	ref
Q2	14.3	3339	45,570	236	1.16	(0.96–1.42)	1.14	(0.93–1.40)
Q3	28.7	3339	45,523	188	1.10	(0.90–1.35)	1.09	(0.87–1.35)
Q4	76.7	3338	45,122	180	0.92	(0.74–1.13)	0.92	(0.74–1.15)
trend *P*						0.084		0.11
Noodles								
Q1	24.8	3339	46,479	181	1.00	ref	1.00	ref
Q2	43.4	3339	45,762	209	1.11	(0.91–1.36)	1.07	(0.87–1.30)
Q3	61.3	3339	45,628	187	1.06	(0.87–1.30)	1.02	(0.83–1.26)
Q4	109.3	3338	45,567	202	1.26	(1.03–1.53)	1.11	(0.90–1.37)
trend *P*						0.034		0.37

HR: hazard ratio, CI: confidence interval. Food intakes were adjusted for total energy intake by Willet method. ^a^ Estimated hazard ratio after adjustments for age (years), marital status (yes, no), education years (≤8 years, 9–11 years, 12–14 years, ≥15 years), body mass index (quartiles), smoking status (never, past, current smoker for 30 years or less, current smoker for 31 years or more), physical activity score, history of diabetes and hypertension (yes, no), alcohol consumption (quartiles), coffee consumption (none, <1, ≥1 cup/day) and salt intake (quartiles).

**Table 4:** Hazard ratios of cardiovascular disease mortality among Japanese women according to the quartiles (Q1–Q4) of intakes of rice, breads, and noodles as grain dishes.

	Median Intake (g/day)	No. of Subjects	Person-Years	No. of Deaths	Age-Adjusted		Multivariate-Adjusted ^a^	
					HR	95% CI	HR	95% CI
Rice								
Q1	127.3	3931	57,360	154	1.00	ref	1.00	ref
Q2	168.0	3931	57,091	217	1.02	(0.83–1.26)	1.00	(0.81–1.23)
Q3	193.2	3931	54,641	365	1.18	(0.97–1.43)	1.11	(0.90–1.36)
Q4	288.7	3931	58,069	171	0.91	(0.73–1.13)	0.87	(0.68–1.11)
trend *P*						0.26		0.18
Bread								
Q1	7.2	3931	57,210	240	1.00	ref	1.00	ref
Q2	19.3	3931	55,605	307	1.20	(1.02–1.43)	1.16	(0.97–1.38)
Q3	37.1	3931	57,191	182	1.09	(0.90–1.32)	1.06	(0.87–1.29)
Q4	78.8	3931	57,154	178	1.03	(0.85–1.25)	0.97	(0.80–1.19)
trend *P*						0.71		0.38
Noodles								
Q1	18.9	3931	56,372	236	1.00	ref	1.00	ref
Q2	32.7	3931	56,276	265	1.07	(0.90–1.27)	1.02	(0.85–1.22)
Q3	45.1	3931	57,142	219	1.13	(0.94–1.36)	1.04	(0.86–1.26)
Q4	76.0	3931	57,370	187	0.98	(0.80–1.18)	0.93	(0.76–1.13)
trend *P*						0.73		0.43

HR: hazard ratio, CI: confidence interval. Food intakes were adjusted for total energy intake by Willet method. ^a^ Estimated hazard ratio after adjustments for age (years), marital status (yes, no), education years (≤8 years, 9–11 years, 12–14 years, ≥15 years), body mass index (quartiles), smoking status (never, past, current smoker for 30 years or less, current smoker for 31 years or more), physical activity score, history of diabetes and hypertension (yes, no), alcohol consumption (quartiles), coffee consumption (none, <1, ≥1 cup/day), salt intake (quartiles) and menopausal status.

**Table 5:** Hazard ratios of mortality from coronary heart disease and stroke among Japanese men according to the quartiles (Q1–Q4) of intake of rice as a grain dish.

	No. of Subjects	No. of Deaths	Age-Adjusted		Multivariate-Adjusted ^a^	
			HR	95% CI	HR	95% CI
Coronary heart disease						
Q1	3339	45	1.00	ref	1.00	ref
Q2	3339	50	1.02	(0.68–1.52)	0.94	(0.62–1.43)
Q3	3339	26	0.72	(0.44–1.18)	0.68	(0.40–1.13)
Q4	3338	33	0.75	(0.48–1.18)	0.63	(0.37–1.07)
trend *P*				0.095		0.046
Stroke						
Q1	3339	104	1.00	ref	1.00	ref
Q2	3339	105	0.90	(0.69–1.18)	0.85	(0.64–1.12)
Q3	3339	51	0.68	(0.48–0.95)	0.74	(0.52–1.05)
Q4	3338	70	0.72	(0.53–0.98)	0.72	(0.51–1.03)
trend *P*				0.009		0.049

HR: hazard ratio, CI: confidence interval. Rice intake was adjusted for total energy intake by Willet method. ^a^ Estimated hazard ratio after adjustments for age (years), marital status (yes, no), education years (≤8 years, 9–11 years, 12–14 years, ≥15 years), body mass index (quartiles), smoking status (never, past, current smoker for 30 years or less, current smoker for 31 years or more), physical activity score, history of diabetes and hypertension (yes, no), alcohol consumption (quartiles), coffee consumption (none, <1, ≥1 cup/day) and salt intake (quartiles).

## Data Availability

The data presented in this study are available on request from the corresponding author. The data are not publicly available due to the privacy of study participants.
